# Transforming discrete choice experiment latent scale values for EQ-5D-3L using the visual analogue scale

**DOI:** 10.1007/s10198-020-01173-0

**Published:** 2020-03-16

**Authors:** Edward J. D. Webb, John O’Dwyer, David Meads, Paul Kind, Penny Wright

**Affiliations:** 1 Leeds Institute of Health Sciences, University of Leeds, Leeds, UK; 2 Leeds Institute of Medical Research At St. James’s, University of Leeds, Leeds, UK

**Keywords:** EQ-5D, Discrete choice experiment, Anchoring, Visual analogue scale, Valuation, I10, I30, D7

## Abstract

**Background:**

Discrete choice experiments (DCEs) are widely used to elicit health state preferences. However, additional information is required to transform values to a scale with dead valued at 0 and full health valued at 1. This paper presents DCE-VAS, an understandable and easy anchoring method with low participant burden based on the visual analogue scale (VAS).

**Methods:**

Responses from 1450 members of the UK general public to a discrete choice experiment (DCE) were analysed using mixed logit models. Latent scale valuations were anchored to a full health = 1, dead = 0 scale using participants’ VAS ratings of three states including the dead. The robustness of results was examined. This included a filtering procedure with the influence each individual respondent had on valuation being calculated, and those whose influence was more than two standard deviations away from the mean excluded.

**Results:**

Coefficients in all models were in the expected direction and statistically significant. Excluding respondents who self-reported not understanding the VAS task did not significantly influence valuation, but excluding a small number who valued 33333 extremely low did. However, after eight respondents were removed via the filtering procedure, valuations were robust to removing other participants.

**Conclusion:**

DCE-VAS is a feasible way of anchoring DCE results to a 0–1 anchored scale with low additional respondent burden.

## Introduction

EQ-5D is a commonly used instrument measuring individuals’ health along five dimensions: mobility (MO), self-care (SC), usual activities (UA), pain and discomfort (PD) and anxiety and depression (AD). In its original, standard format each dimension has either three response levels (no problems, some problems, extreme problems) Health state 12132, for example, indicates an individual has mobility measured at level 1, self-care measured at level 2, etc. When used to calculate QALYs in the economic evaluation of health interventions a value of 1 is assigned to full health (i.e., state 11111) with the value of dead set to 0. All other EQ-5D health states are valued on a full health = 1, dead = 0 scale with health states regarded as being worse than dead having negative values. A similar format can be found in other generic measures used to compute QALYs, for example, HUI [1] and SF-6D [2]. Several different methods have been used to determine values for use in these instruments, including time trade-off (TTO) and standard gamble (SG). Individuals either sacrifice life-years (TTO) or accept a risk of instant death (SG) to improve their health to full health, from which the value of a given health state can be inferred.

Both TTO and SG have been criticised due to the difficulty of understanding them and the cognitive burden they place on participants [3–5]. In addition, methods which involve trading life-years or risking instant death can be inappropriate if used to value mild health states [6], or for other reasons [7] discussed later.

Discrete choice experiments (DCEs) are a popular tool in health to elicit preferences [8, 9], and recently there has been interest in using them to value EQ-5D [10–20]. DCEs present individuals with two or more options, each described in terms of a set of attributes. Individuals then simply choose which one they prefer. They make several choices with the levels of attributes varied in each question, and from the responses, relative preferences for the levels of each attribute can be estimated.

DCEs are relatively easy to understand, and the response mode is simple and direct. The cognitive burden of TTO and SG means that they are usually interview-administered, whereas DCEs can be readily self-completed, reducing cost. It is also possible to find preferences for a large number of hypothetical objects by presenting only a subset of them [21]. A disadvantage of DCEs is that results are on a latent scale: the resulting preferences are measured in terms of unanchored utility with no units. Thus some means of anchoring results to a full health = 1 and dead = 0 scale is required if the value sets are to be used to construct QALYs. At present there is no standard way of anchoring DCE results.

An exhaustive list of techniques to anchor latent scale DCE valuations is beyond the scope of this paper, however, three common approaches will be discussed. The first is termed DCE_TTO_ (also known as DCE with duration), in which a sixth attribute (duration) is added to the options respondents choose between. Respondents are instructed to imagine living in health states for the specified number of years, followed by immediate death. The time dimension gives information on how preferred a state is relative to dead, and thus makes a transformation to the 0–1 scale possible. Examples are Viney et al. [15] and Bansback et al. [19].

Another method is to elicit preferences for several health states using TTO as well as the DCE. The data from both are combined and a hybrid statistical model estimated. Information from the TTO tasks ensures DCE valuations from the hybrid model are on a 0–1 scale. Examples are Devlin et al. [10] and Ramos-Goñi et al. [16]. The EQ-VT protocol recommended by the EuroQol group to construct EQ-5D-5L value sets uses such a hybrid method.^1^

Finally, some studies use a dual response design. Participants are either presented with two EQ-5D states and the state dead and asked to choose their most and least preferred state, or asked to choose between two EQ-5D states then subsequently asked whether their chosen state is better or worse than being dead. Preferences for EQ-5D states can be measured relative to dead and results transformed to be on a 0–1 scale. Examples include Ramos-Goñi et al. [18] and the general population sample in Stolk et al. [20]. It should be noted that such designs have been criticised on theoretical grounds [22, 23].

Some studies use a combination of methods, for example Viney et al. [15] include duration as an attribute as well as including dead as a choice.

In this paper, another approach to anchoring DCE results valuing EQ-5D-3L to a 0–1 scale is outlined, which is termed DCE-VAS. It anchors results using responses to a single visual analogue scale (VAS) task in which respondents assign a value between 0 and 100 to three health states: 11111, 33333 and dead. It (1) is easy to understand; (2) imposes a light burden on participants; and (3) is simple to implement online. Although EQ-5D-3L value sets have been constructed using VAS in the past (e.g., [24–26].), this is the first study of which we are aware to combine DCE and VAS.

This paper examines the viability of using DCE-VAS to construct a value set for the EQ-5D-3L, and discusses its advantages and disadvantages relative to alternatives.

## Methods

### Survey

**Fig. 1:**
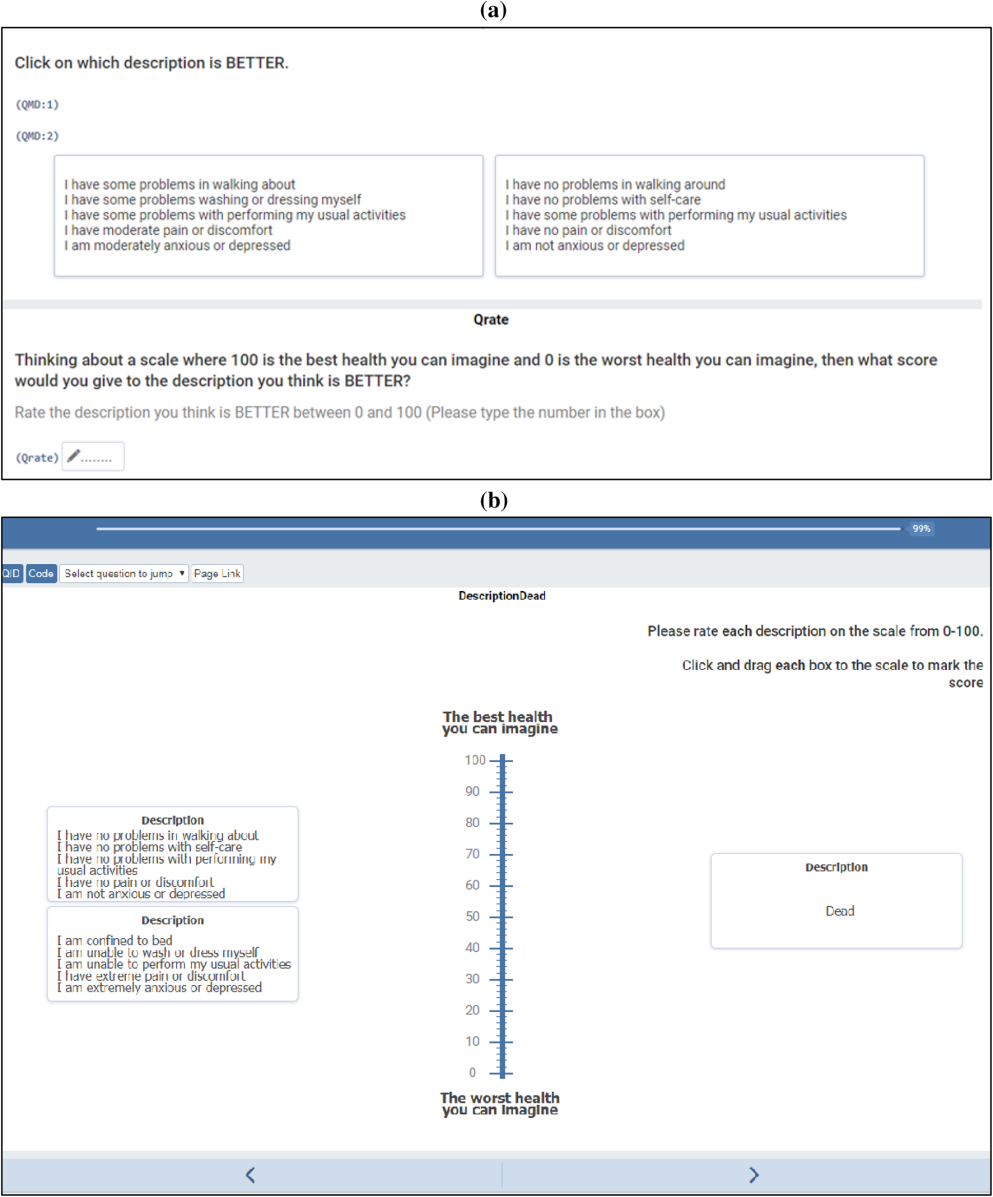
Example visual analogue scale task

Participants began by answering some questions about themselves (age, gender, etc.) and their health, including self-reported EQ-5D. They then completed a DCE in which they indicated which of two EQ-5D health-states they considered to be better. They were then asked to score their chosen health state on a scale where 100 was the best and 0 the worst health they could imagine. (Note this is not a VAS as no thermometer was presented.) The DCE had a D-efficient design generated using the choiceDes package for R with 40 tasks split into four blocks. An example task is shown in Fig. 1a. Our recruitment target was ample to estimate a main effects model according to several sample size rules of thumb [21, 27, 28], as well as being in line with DCE sample sizes for similar studies [8]. At the end of the DCE, participants completed one further task presenting a dominated choice (12121 vs. 23232). Participants who chose the dominated choice in this task were considered to have failed the dominance test.

Participants also valued several health states using an online adaptation of the EQ-VAS (see Fig. 1b). A thermometer scale was displayed vertically on the screen with values from 0–100. 0 was described as “the worst health you can imagine” and 100 was described as “the best health you can imagine”. Health states were displayed in boxes which participants dragged to place on the scale. The precise value selected was indicated by a line drawn from the box to the scale and a number displayed in the corner of the box. All participants performed two VAS tasks: (i) rating their own health that day and (ii) rating 11111, 33333 and dead. This presentation was based on the method of Kind, Hennessy and Macran [29], who presented all four at once. However, in the present study, so as to minimise ethical issues around judging if one’s own health was better or worse than dead, own health was presented on a separate screen.

Once participants had completed the final VAS task, they were asked the following question: *“*Which of these best describes your thoughts about the final health rating question? (i.e., rating of the three descriptions)?*”* with possible responses: *“*I completely understood what I was supposed to do*”*; *“*I think I understood what I was asked to do*”*; *“*I do not think I understood what I was asked to do*”*; and “I did not understand what I was asked to do*”*. An understanding rating for each participant was formed by coding responses as 1 = completely understood, 2 = thought they understood, etc. Thus a lower rating implies greater understanding of the task.

**Fig. 2:**
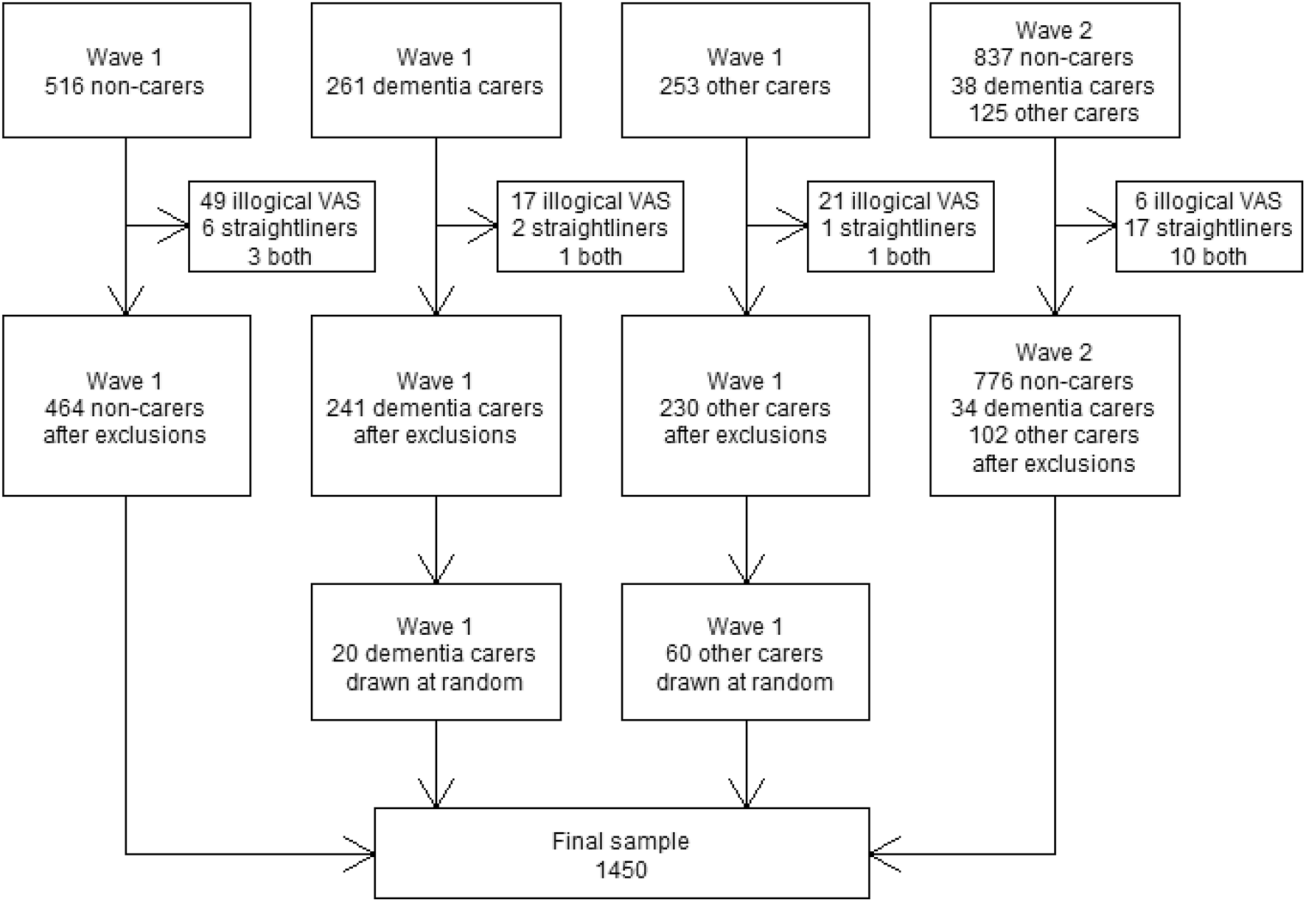
Flow diagram illustrating the selection of the final sample for analysis

The survey was administered online for ease of recruitment and participants were recruited from an online panel managed by a survey and market research company. Data were collected in two waves. In the first, carers for people with dementia and carers for people with other conditions were oversampled due to the requirements of a separate research project, whereas in the second, there was no oversampling. To correct for this, the proportion of both carer groups in the second wave was calculated, and a corresponding fraction drawn at random from carer groups in the first waves to include, with the rest of the responses from those groups discarded. Figure 2 illustrates how the final sample size was arrived at.

## Analysis

DCE responses were analysed using mixed logit models of the form

$$u_{ij} = 1 + \mathop \sum \limits_{k} \beta_{ik} x_{ijk} + \varepsilon_{ij}$$

where ${u}_{ij}$ is the utility to person *i* of option *j*, ${x}_{ijk}$ are a set of dummy variables for all level 2 and level 3 states and ${\varepsilon }_{ij}$ is an error term. ${\beta }_{ik}$ are coefficients which measure the decrement in utility associated with a level 2 or 3 state compared to a level 1 state. The constant 1 normalises the utility of full health (11111) to be 1. Coefficients were modelled as normally distributed with mean ${\beta }_{k}$ and variance ${\sigma }_{k}^{2}$. Mixed and multinomial logit models are the most commonly used models in health DCEs [8]. Mixed logit models were preferred as they may provide more accurate parameter estimates due to accounting for preference heterogeneity [30, 31].

Raw VAS valuations of health states were anchored to be on a full health = 1, dead = 0 scale using the equation

1 $$\widetilde{{{\text{VAS}}}}_{i} = \frac{{{\text{VAS}}_{i} - {\text{VAS}}_{{{\text{dead}}}} }}{{{\text{VAS}}_{{11111}} - {\text{VAS}}_{{{\text{dead}}}} }}$$

where ${\mathrm{V}\mathrm{A}\mathrm{S}}_{i}$ and ${\widetilde{{\mathrm{V}\mathrm{A}\mathrm{S}}}_{i}}$ are, respectively, the raw and anchored VAS valuations of state *i*.

Results from mixed logit estimation are on a latent scale. Coefficients were transformed according to

2 $$\tilde{\beta }_{k} = \left( {\frac{{1 - \widetilde{{{\text{VAS}}}}_{33333} }}{{\mathop \sum \nolimits_{j = 1}^{5} \beta_{j3} }}} \right)\beta_{k}$$

where ${\tilde{{\beta}}}_{k}$ is the rescaled coefficient and ${\beta }_{j3}$ are the latent scale level 3 coefficients. The valuation of some EQ-5D state *j* on the 0–1 scale, ${\tilde{{u}}}_{j}$ is finally

$$\tilde{u}_{j} = 1 + \mathop \sum \limits_{k} \tilde{\beta }_{k} x_{jk}$$

**Fig. 3:**
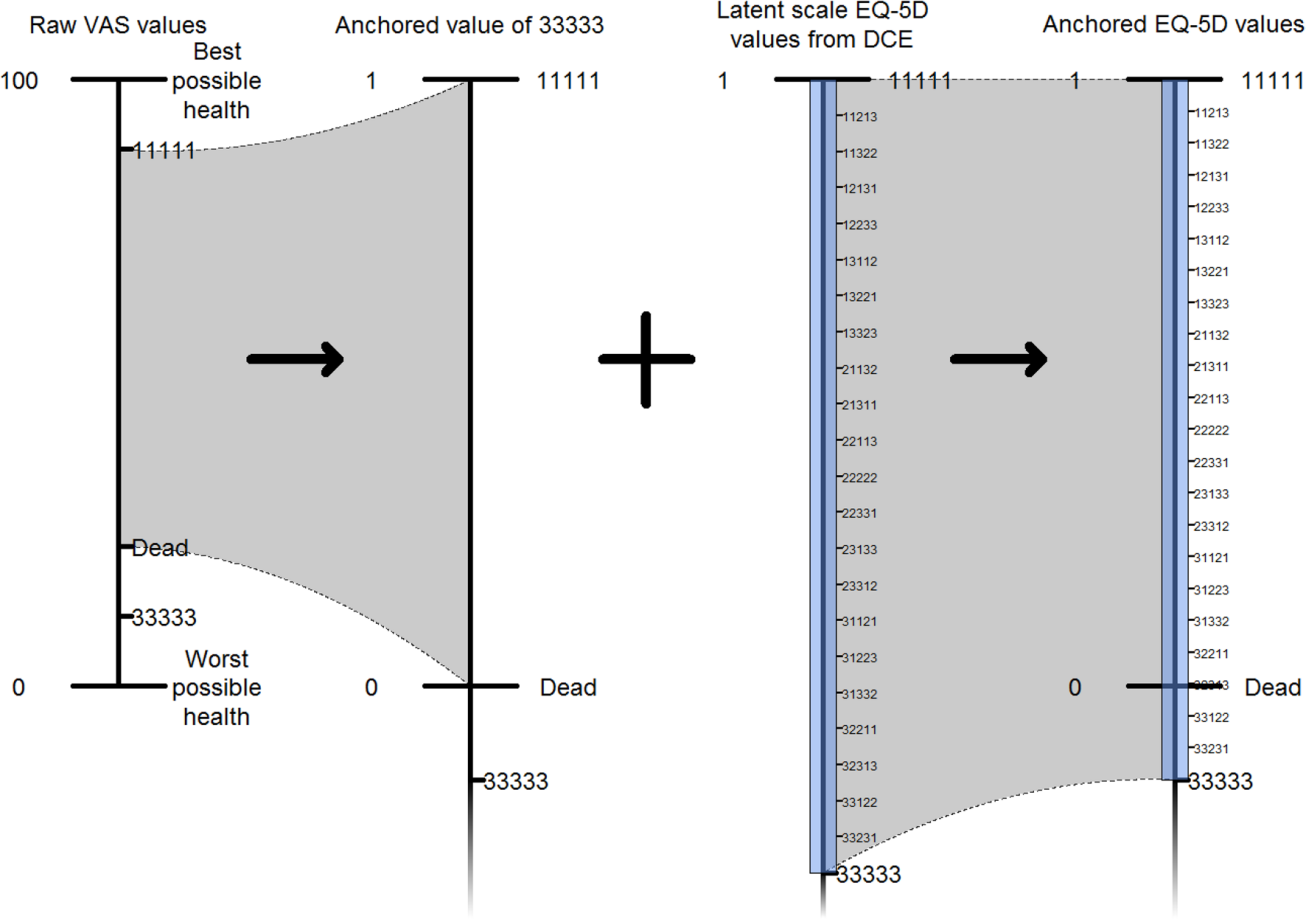
Visual illustration of anchoring method

Figure 3 gives a visual illustration of the anchoring method. The left-hand side illustrates how an anchored value of 33333 is obtained (Eq. (1)) and the right-hand side illustrates the transformation of latent scale health state valuations from the DCE to anchored valuations.

In order for the anchoring procedure to give meaningful results, participants needed to give logical answers to the VAS, i.e. ${\mathrm{V}\mathrm{A}\mathrm{S}}_{11111}>{\mathrm{V}\mathrm{A}\mathrm{S}}_{33333}$ and ${\mathrm{V}\mathrm{A}\mathrm{S}}_{11111}>{\mathrm{V}\mathrm{A}\mathrm{S}}_{\mathrm{d}\mathrm{e}\mathrm{a}\mathrm{d}}$. Thus all participants giving illogical VAS answers were removed from the data prior to analysis, as well as participants who “straight-lined” DCE answers, i.e. always chose the same option.

Mixed logit models of the form outlined above were estimated using the following samples:-

**Table:** 

(i)	ALL:	Full sample for analysis
(ii)	DOM:	Participants who failed the dominance test removed
(iii)	U4:	Participants with an understanding rating of 4 removed
(iv)	U3:	Participants with an understanding rating of 3 or 4 removed
(v)	U2:	Participants with an understanding rating of 2, 3 or 4 removed
(vi)	D50:	Participants with ${\mathrm{V}\mathrm{A}\mathrm{S}}_{\mathrm{d}\mathrm{e}\mathrm{a}\mathrm{d}}>50$ removed
(vii)	D20:	Participants with ${\mathrm{V}\mathrm{A}\mathrm{S}}_{\mathrm{d}\mathrm{e}\mathrm{a}\mathrm{d}}>20$ removed
(viii)	D10:	Participants with ${\mathrm{V}\mathrm{A}\mathrm{S}}_{\mathrm{d}\mathrm{e}\mathrm{a}\mathrm{d}}>10$ removed

Anchored coefficients for models (ii)–(viii) were converted to be expressed as percentages of the magnitude of the coefficients from model (i).

The anchoring process involves combining data from participants who regard 33333 as worse than dead and those who do not. To examine whether these groups differed in other ways in their valuation of health states, separate MIXL models were estimated for each and Welch’s *t* test used to test for differences in (unanchored) coefficients.

The sample was also refined using a procedure designed to ensure that no individual respondent has an excess influence on results. A series of multinomial logit (MNL) models were run with each respondent excluded from the sample in turn and the results anchored to the full health = 1, dead = 0 scale. The magnitude of coefficients was calculated as a fraction of the coefficients from an MNL model with the full sample to find the individual’s average influence on results. The overall mean of individuals’ influence on results was calculated and respondents whose influence on results was more than two standard deviations away from this excluded. Finally, a MIXL model with the resulting sample was run and termed FILTER.

For ALL, *t* tests were performed on the null hypothesis that mean level 2 coefficients were equal to 0 and that level 2 and level 3 coefficients were equal. For all other models, *t* tests were performed on the null hypothesis that coefficients expressed as a percentage of ALL coefficients were equal to 100%. Significance was judged at the 5% level adjusted for multiple testing using Holm’s sequential Bonferoni correction [32]. EQ-5D value sets were constructed for each sample in (i)–(viii) using Eq. (1).

A panel random-effects linear model was estimated using maximum likelihood with the rating of preferred health states out of 100 as the dependent variable and EQ-5D levels as independent variables. The resulting coefficients were used to predict ratings for all possible health states and the intraclass correlation coefficient (ICC) between these and the FILTER value set calculated.

All analysis was carried out using R version 3.3.1 with logit models estimated using the CMC Choice Modelling Centre Code for R version 1.1 [33].

## Results

A total of 2030 participants completed the survey, out of which 1450 were used for analysis (see Fig. 2).

Table 1 contains summary statistics of sample demographics. The mean age was around 46. The proportion of women was 53%, and a large majority of participants (89%) were of white ethnicity. 54% were employed or self-employed, and just over 20% were retired. The sample was generally well educated, with around half having a degree or equivalent professional qualification, and three-quarters leaving school after the minimum age.

**Table 1:** Summary statistics of participants' demographics and VAS responses

Age	Mean	45.8
	Standard deviation	17.1
	Min	18
	Max	92
Female (%)		52.9
White (%)		89.4
Occupation	Employed/self-employed	53.7
	Retired	21.4
	Housework	6.48
	Student	7.03
	Unemployed	5.38
	Other	6
Education	Left school after minimum age	75.4
	Degree/professional qualification	51
Dependents	Children < 18 in household	25.9
	Mean no. of children	1.92
VAS	Own health mean	74.2
	Standard deviation	18.7
	11111 mean	92.4
	Standard deviation	12.9
	33333 mean	19.2
	Standard deviation	16.9
	Dead mean	7.11
	Standard deviation	14.9
Vas understanding rating (%)	1	59.9
	2	37
	3	2.34
	4	0.69
*N*		1450

Table 1 also contains average participant VAS ratings. As expected, 11111 is rated the highest (mean 92) and 33333 is rated higher than dead (19 vs 7). The mean rating of participants’ own health was 74. Very few participants had a VAS understanding rating of 3 or 4 (i.e., stated they didn’t or didn’t think they understood the task), however, 37% had a rating of 2 (i.e. they stated they only thought they understood the task rather than being certain).

Table 2 contains the rescaled coefficients of all models. For ALL, all coefficients are significant and in the expected direction, i.e. level 2 coefficients are negative, level 3 coefficients are lower than level 2. The dimension with the greatest impact was mobility (− 0.09 for level 2, − 0.40 for level 3) whereas the least impact was for usual activities (− 0.05 for level 2, − 0.12 for level 3). The magnitude of coefficients for DOM is between 94.5% and 108% of the magnitude of ALL coefficients, however, these differences are not statistically significant. The magnitudes of coefficients of U2, U3 and U4 are typically around 95% of those from ALL, but again the differences are not significant. Significant differences are seen for almost all coefficients in D50, D20 and D10. All coefficients were of lower magnitude than those of ALL, with differences typically being around 70–75%.

**Table 2:** Utility decrements for each EQ-5D-3L dimension

	ALL	DOM	U4	U3	U2	D50	D20	D10	FILTER
MO2	− 0.0863*	96.8	94	94	93.4	73.3*	70.5*	70.6*	80.2*
	[− 0.0981, − 0.0744]	[83.9, 109.7]	[81.0, 107.0]	[81.0, 107.0]	[80.5, 106.3]	[63.0, 83.6]	[60.3, 80.7]	[60.1, 81.1]	[69.1, 91.3]
MO3	− 0.398*	94.5	95.6	96	95.4	74.8*	72.5*	71.1*	80.6*
	[− 0.425, − 0.37]	[87.3, 101.6]	[89.0, 102.2]	[89.1, 102.8]	[88.6, 102.2]	[69.5, 80.0]	[67.3, 77.8]	[65.7, 76.5]	[75.1, 86.2]
SC2	− 0.0731*	103.6	94.7	95.7	95.1	75.6*	76.6*	76.3*	80.8*
	[− 0.0849, − 0.0613]	[88.8, 118.4]	[79.3, 110.2]	[80.1, 111.2]	[79.7, 110.5]	[63.8, 87.5]	[64.4, 88.7]	[63.7, 88.8]	[67.9, 93.6]
SC3	− 0.166*	98.5	95.2	96.5	95.9	74.8*	73.6*	72.3*	80.1*
	[− 0.186, − 0.146]	[86.5, 110.5]	[83.4, 107.0]	[84.7, 108.2]	[84.2, 107.6]	[65.6, 84.1]	[64.3, 82.9]	[62.7, 81.9]	[70.4, 89.8]
UA2	− 0.0537*	104.3	97.6	96.9	96.3	78.4*	82.7	89.6	82.2
	[− 0.0672, − 0.0401]	[81.1, 127.6]	[73.6, 121.6]	[73.0, 120.7]	[72.6, 120.0]	[59.7, 97.0]	[64.2, 101.2]	[71.0, 108.2]	[62.0, 102.4]
UA3	− 0.122*	104.1	96.3	95.5	94.9	76.7*	75.3*	76.0*	81.2*
	[− 0.138, − 0.106]	[91.3, 116.8]	[83.9, 108.7]	[83.1, 107.8]	[82.7, 107.2]	[66.9, 86.5]	[65.5, 85.1]	[65.9, 86.1]	[70.7, 91.6]
PD2	− 0.0831*	105.3	96.1	96.7	96.2	76.0*	74.2*	74.5*	80.3*
	[− 0.0942, − 0.0719]	[93.0, 117.6]	[83.3, 108.9]	[83.9, 109.6]	[83.4, 108.9]	[65.9, 86.1]	[64.1, 84.2]	[64.2, 84.7]	[69.5, 91.0]
PD3	− 0.24*	102.5	95.4	96.2	95.6	75.3*	72.7*	71.2*	80.1*
	[− 0.259, − 0.221]	[94.4, 110.6]	[87.8, 102.9]	[88.5, 103.9]	[88.0, 103.3]	[69.1, 81.5]	[66.6, 78.8]	[64.9, 77.4]	[73.7, 86.5]
AD2	− 0.0725*	108.1	95.3	96.4	95.8	74.5*	72.5*	73.1*	80.2*
	[− 0.0834, − 0.0616]	[94.3, 122.0]	[80.9, 109.7]	[82.2, 110.6]	[81.7, 109.9]	[63.2, 85.8]	[61.5, 83.5]	[61.6, 84.6]	[68.1, 92.3]
AD3	− 0.193*	102.3	94.4	95.3	94.8	74.2*	70.5*	69.8*	79.2*
	[− 0.21, − 0.176]	[93.4, 111.2]	[85.9, 102.9]	[86.8, 103.9]	[86.3, 103.3]	[67.4, 81.0]	[63.8, 77.1]	[62.8, 76.8]	[72.0, 86.4]
Value of 33333	− 0.119	− 0.110	− 0.067	− 0.074	− 0.067	0.61	0.187	0.199	0.102
*N*	1450	1287	1440	1406	869	1389	1276	1115	1442

Models other than ALL expressed as percentages

*Indicates significance at the 5% level of *t* tests of equality of level 2 coefficients to 0 and equality of level 2 and 3 coefficients (ALL) and equality of coefficients to 100% (all other models). 95% confidence intervals in [ ]

Table 3 gives the latent scale coefficients for models estimated separately for participants who did and did not value 33333 as worse than dead. No significant differences were observed between the two groups.

**Table 3:** Latent scale coefficients for mixed logit models estimated separately for participants valuing dead worse than 33333 on VAS task and participants valuing 33333 worse than dead

	Dead worse than 33333	Standard error	33333 worse than dead	Standard error	*p* value
MO2	− 0.579	0.0463	− 0.547	0.0836	0.735
MO3	− 2.75	0.111	− 2.43	0.184	0.143
SC2	− 0.508	0.0466	− 0.451	0.0801	0.536
SC3	− 1.08	0.0792	− 1.22	0.142	0.389
UA2	− 0.382	0.0535	− 0.298	0.0921	0.428
UA3	− 0.832	0.0634	− 0.754	0.106	0.529
PD2	− 0.564	0.0437	− 0.553	0.0788	0.899
PD3	− 1.57	0.0742	− 1.69	0.137	0.445
AD2	− 0.526	0.0426	− 0.338	0.0732	0.026
AD3	− 1.26	0.0656	− 1.32	0.129	0.707
*N*	1103		347		

*p* values for Welch’s *t* test

*Indicates significance at the 5% level

All coefficients in the FILTER model are of lower magnitude than for ALL, with the exception of usual activities level 2, with values typically around 80%.

**Fig. 4:**
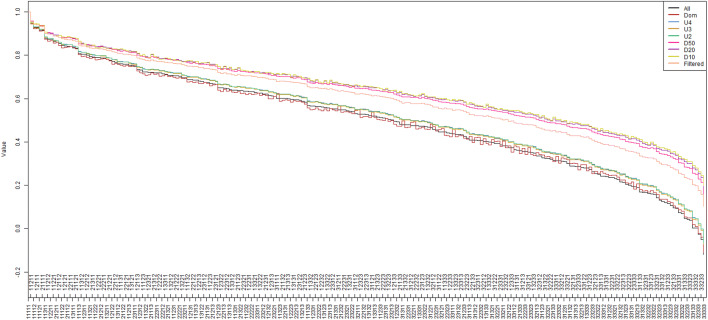
Comparison of value sets

Figure 4 compares the value sets from all models. Value sets from ALL, DOM, U4, U3 and U2 are similar. Value sets from D50, D20 and D10 are also similar to each other, but with generally higher valuations. FILTER lies in between these two groups.

**Fig. 5:**
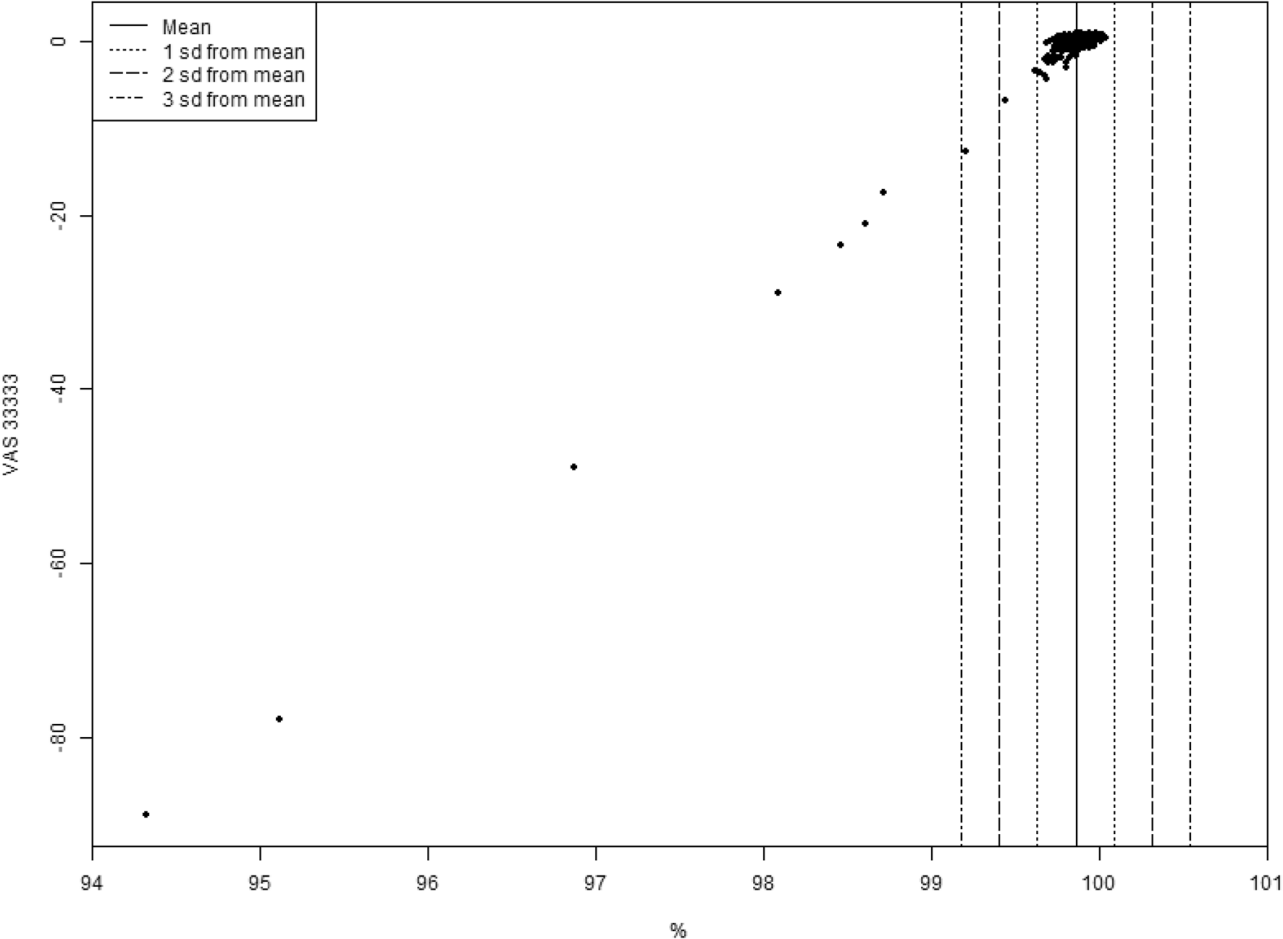
Correlation between anchored VAS valuation of 33333 and mean MNL coefficients estimated excluding a single respondent expressed as a percentage of mean MNL coefficients with all respondents

Table 4 lists how many participants who were excluded from the FILTER sample were also excluded from other models’ samples. Eight common participants were excluded from FILTER, as well as D50, D20 and D10, whereas there were fewer commonly excluded participants with FILTER, and DOM, U4, U3, and U2. Table 5 lists the VAS responses for all participants excluded from the FILTER model. All had extremely low anchored valuations of 33333, generally due to rating both 11111 and dead highly. This is confirmed by Fig. 5, which shows the correlation between participants’ anchored VAS valuations of dead and their influence on valuation.

**Table 4:** Number of respondents excluded in FILTER model excluded in other models

Model	*N*
DOM	2
U4	1
U3	1
U2	4
D50	8
D20	8
D10	8

**Table 5:** VAS responses from each participant excluded from FILTER model

Anchored VAS 33333	VAS 11111	VAS 33333	VAS dead
− 89	100	10	99
− 78	91	12	90
− 49	100	0	98
− 29	100	10	97
− 23.5	98	0	94
− 21	88	44	86
− 17.3	94	39	91
− 12.7	61	20	58

**Fig. 6:**
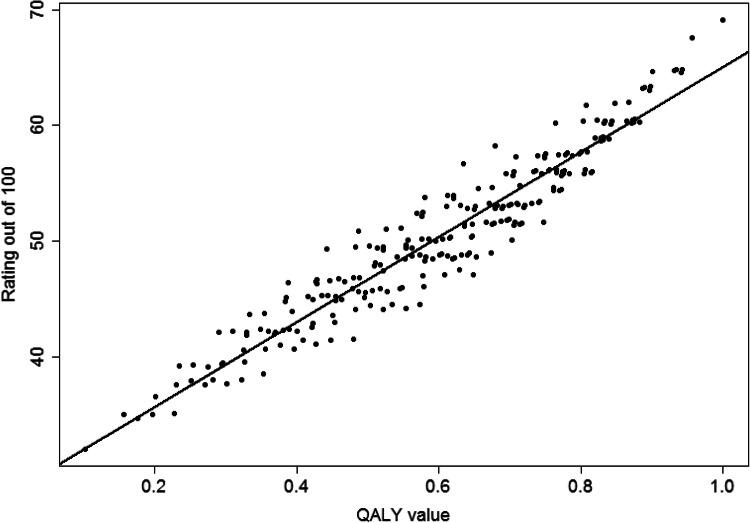
Correlation between FILTER value set and predicted rating of states from random effects model

Figure 6 compares the value set from FILTER and ratings out of 100 for each state predicted from the panel random effects logit model. The ICC is 0.96, which is generally accepted to represent excellent agreement [10, 34].

## Discussion

This paper has presented an alternative to existing methods and demonstrated that it is possible to use DCE-VAS to construct value sets that are logically consistent and plausible. The properties of the valuations obtained are discussed, followed by comparing DCE-VAS to alternatives.

From Table 2 it can be seen that excluding respondents who failed various measures of response quality left valuations largely unchanged, as there were no significant differences between the results from ALL and DOM, U4, U3 and U2. It is thus reassuring that results are not particularly dependent on those who may not have attended to the tasks or failed to understand them. However, it is important to note that self-assessed understanding of a task may not be a good measure of those who actually understand it: those who fail to grasp a task may also fail to grasp their lack of understanding [35]. Greater differences, which in every instance are statistically significant, are seen when individuals who gave a high VAS ranking for dead are excluded. However, note there is little difference in the magnitude of anchored coefficients when removing the 61 (4.2%) of respondents who rated dead above 50 and when removing the 335 (23.1%) who rated it above 10.

The reason why may be gleaned by examining the results of the FILTER model, which are similar to D50, D20 and D10. Eight participants had a large amount of influence on valuation, defined as more than two times the standard deviation of the mean influence of all participants. As can be seen in Table 4, all eight of these were also excluded from the D50, D20 and D10 models, yet no more than four were also excluded from DOM, U4, U3 or U2. Table 5 lists the VAS responses of all participants excluded from the FILTER model, and it is apparent that the reason for having a large influence is that their VAS valuations of 33333 on the full health = 1, dead = 0 scale are extremely low, at between − 89 and − 12.7. This leads to their valuations having a large impact on the mean valuation of 33333 and in turn on the scale factor used to anchor latent scale coefficients.

The recommendation for future studies using DCE-VAS is to examine whether participants with extremely low VAS valuations of 33333 have an excess influence on results, and if so to use the FILTER procedure to obtain the final results. This recommendation is made on several grounds. First, it removes only a small number of respondents, only 0.55% of the sample for analysis in this case. Second, it appears that the results of the FILTER model are robust to further removal of individuals giving high VAS ratings for dead. It is also grounded in a philosophically defensible principle: that if EQ-5D value sets are to represent the views of a population, they should not be overly influenced by any single individual survey respondent. Finally, although the threshold of two standard deviations away from the mean influence is arbitrary, it may be seen from Fig. 5 that the thresholds may be varied considerably without changing the number of excluded respondents significantly.

From Table 5, most of the excluded respondents gave very similar, high ratings to both 11111 and dead. It may be that they misunderstood the task, yet by chance submitted logically consistent values. However, it is also possible that the respondents genuinely viewed the state 33333 as so bad that dead should be assigned a relatively high value in comparison. In this case, it is a limitation of the method that such legitimate views are excluded. However, note that such extremely low valuations of states are also generally explicitly or implicitly excluded or censored in other methods of anchoring DCE data. For example, in the EQ-VT protocol, the lowest value it is possible for a state to have is − 1.

The value sets in this paper were constructed by simply taking the mean of all participants’ VAS valuation of 33333, which combines the views of heterogeneous groups. In particular, it combines the views of those who regard 33333 as better than dead and those who regard it as worse. This raises concerns that individuals with differing attitudes towards dead may have differing attitudes towards other aspects of health. However, when separate models were estimated for each group, no significant differences were found, which suggests their views on the relative severity of EQ-5D dimensions are reasonably similar. Nevertheless, the opinions of many individuals are amalgamated in the models presented here, and future research could usefully examine methods such as latent class analysis which allows for heterogeneity between groups.

The value of 33333 was somewhat greater than dead in the FILTER model, in contrast to many other EQ-5D-3L value sets (for example 33333 is valued at − 0.594 in the MVH value set for the UK [36]). However, it is not uncommon for VAS values to be higher than TTO or SG based values, and previous studies have found VAS values of 33333 similar to or greater than dead. For example Gudex et al. [26] found a mean anchored value of 0.00, and Johnson et al. [37] present two valuation exercises with mean raw values of 15.82 and 17.55 for 33333 compared to 13.38 and 14.82 for dead.

In principle, any EQ-5D-3L state could have been included in the VAS task alongside 11111 and dead (other than 11111 itself). The state 33333 was chosen as it showed directly whether an individual regarded any state as worse than dead, and the range EQ-5D-3L occupies on the full health = 1, dead = 0 scale. It would be useful in future work to examine the effect of anchoring intermediate states, and whether it would improve the validation of anchoring, especially for respondents who assigned very low values to 33333. A potential disadvantage of this approach is that including additional states would increase respondent burden.

Much of the following discussion relates to the properties of VAS relative to alternative techniques. There is an extensive literature on this topic (e.g. [5, 38–40].), and summarising it in its entirety is beyond the scope of this paper. Rather, only issues which are especially relevant for the current study are touched on.

An explicit duration was not indicated for health states, either in the DCE or VAS components, in contrast to many other studies. A potential objection is that health only exists if it is experienced over a finite time, and thus comparing health states without duration is illogical. However, not specifying a specific duration does not mean that it is impossible to imagine occupying a given health state. It is possible to imagine, for example, having a different job, or living somewhere else, without specifying how long one would be employed, or how long one would live there.

From one point of view “dead” does have a duration, in contrast to the other two states in the VAS task, which may lead to conceptual problems in comparing them. On the other hand, in terms of the experience of a given health state, it could be argued that dead is unique in being without a duration: those occupying the state do not experience occupying it for any length of time (discounting religious beliefs). Ultimately, the best defence to such concerns is practical: the vast majority of participants were able to give coherent, logical responses to the VAS task.

Not including duration may cause other problems. Participants may have assumed that a poor health state implies a shorter life expectancy than a good health state, and taken these inferred durations into account when choosing. It is not possible to address this problem with the available data, and it would be a useful topic for future research.

Including duration as an attribute can also cause practical and theoretical problems. Calculating health-state valuations using either DCE_TTO_ or a hybrid model requires making the strong assumption that the valuation of that state is independent of the time spent in it. Thus the utility of spending a year in a given health-state is the same regardless of whether it is followed by another 10 years in it, immediate death, or 10 years in full health. However, these are a large body of evidence that individuals’ perception and experience of time is non-linear in many ways (see e.g. [41].). There is also evidence that the trajectory of expected future health (e.g. improving or deteriorating) can influence valuation [42–44]. Finally, a practical problem is introduced that including duration can make prospects unrealistic for many people. Younger people expecting to live for 50–60 more years may find it difficult to imagine dying in one or two years, while other respondents may find the prospect of living for another decade or more unrealistic.

Comparing DCE-VAS to existing methods, a major advantage is its simplicity and low participant burden. Participants must complete only one additional task, valuing three states in addition to the DCE questions, and surveys using it may be easily administered online. In contrast, hybrid methods typically ask respondents to complete several additional tasks (10 in both Ramos-Goñi et al. [16] and Devlin et al. [10]) using the more complicated TTO methodology. Indeed, each TTO task consists of several responses before the point of indifference is identified, and hybrid methods must also include “lead time” to account for the possibility that respondents may value a state lower than dead. The cognitive difficulty of TTO thus leads some studies (e.g. [10, 16].) to use a more labour intensive face-to-face mode of administration, rather than online. Using VAS, on the other hand, means those who value 33333 as worse than dead can be accommodated just as easily as those who value it as better.

DCE_TTO_ requires no additional task, however, the addition of the duration dimension makes the DCE tasks themselves more cognitively demanding. This is not just due to the extra attribute, but also as it involves an extra feat of imagination to consider not just being in a given health state, but the prospect of occupying it for a set number of years followed by immediate death. As mentioned above, this may also make choices unrealistic for young or very old respondents. In contrast, without explicit duration the choice is a more intuitive and straightforward one of which state would be more preferable to be in, albeit with the aforementioned problem of more severe states being associated with shorter duration.

Valuation of EQ-5D has usually focused on choice-based methods, in line with guidelines from e.g. the National Institute for Clinical Excellence (NICE) in the UK [45]. While the DCE component of DCE-VAS is choice based, it is debateable to what extent the VAS task is. The VAS task here requires simultaneously rating three health states. Participants must choose whether to rank 33333 as better or worse than dead, however, this alone is simply a ranking, which does not give enough information to anchor DCE values. Participants must also choose how far apart to place the states, so the issue becomes whether such choices give meaningful information about participants’ strength of preference for health-states. A detailed analysis of this issue is beyond the scope of this paper. However, there is evidence that, while there are issues with raw VAS responses [46], values that are transformed using similar methods to those used here are capable of measuring the strength of preference [47, 48].

A concern is also that VAS does not capture opportunity cost [49], as participants do not have to sacrifice (expected) life-years in exchange for better health. Yet this argument relies on an (often unspoken) assumption that individuals are willing to trade life-years, either with certainty or in expectation, solely due to their health-related quality of life. Consider an individual who is unwilling for religious reasons to trade any length of life for improvement from 33333 to 11111. It does not follow that the individual regards the health-related quality of life of both states to be equal.

There are many other reasons that the length of (expected) life individuals are willing to trade may not represent their opinion about health-states’ relative quality of life. These include not wanting to be a burden on others, and not wanting to risk death due to responsibilities, for example as a carer [7]. There is growing interest in measuring health spillovers [50], especially for carers [51], so using VAS-based methods may be advantageous in these areas.

Even if the VAS task is accepted as choice-based, an argument could be made that it is still not suitable for creating value sets for QALY calculation since it does not explicitly embody opportunity cost, as DCE_TTO_ and hybrid methods do. However, while optimal economic allocation of resources unarguably requires opportunity costs to be taken into account, it is not clear why this should be the case in simply ascribing values to health states. Indeed, it is not clear why choice-based methods and only choice-based methods should be used in valuation.

The ease and accessibility of DCE-VAS are desirable attributes for a method to have. Values of health states from the general public are used in economic evaluation on the principle that the public’s preferences should direct the allocation of the public’s responses. Thus, it is important for elicitation methods to avoid excessive demands in terms of time and cognitive ability, as this will inevitably exclude certain sections of society. Finally, models based on DCE-VAS are relatively easy to estimate econometrically, in contrast to most hybrid approaches, requiring very little more than standard DCE analysis tools. This means more complicated model specifications, for example introducing interaction terms, can readily be estimated.

The study itself has some weaknesses. It was designed to compare the valuations of carers to the general population, and as such was not specifically designed to test the DCE-VAS methodology. Thus for example, we do not directly compare it to any other method, nor do we collect measures of participant burden or understanding, baring a single question on the VAS task.

We used a survey and market research company to recruit our sample. As a matter of standard practice they removed responses from participants who completed the survey sufficiently quickly they were deemed to not be engaging meaningfully with it prior to providing the data to the research team. As no information is hence available on these removed respondents, it is difficult to tell whether the procedure systematically biases the data. Another disadvantage to this method of recruitment is that some individuals may be “professional” respondents: those who answer a large number of surveys, and whose responses are not typical of the general public.

The sample differs in some respects from the UK population. Almost half the sample had a degree or equivalent, which raises concerns that the survey might perform less well in eliciting the values of a less educated sample. However, it should be noted that almost a quarter of the analysis sample left school at the minimum age.

The study also has many strengths. We estimated models using advanced modelling techniques, and assessed the robustness of results in several ways, including comparing estimation methods. We examined not only the statistical significance of coefficients in econometric models but also compared the EQ-5D value sets constructed from them. Thus the potential implications of using these value sets have been illustrated.

## Conclusion

DCEs are an excellent method for easily eliciting individuals’ valuations of healthcare survey instruments. However, additional information is required to anchor the results to a fixed scale.

This paper contributes by demonstrating the feasibility of using a single VAS task to produce an EQ-5D-3L value set on the full health = 1, dead = 0 scale. However, the claim that DCE-VAS is superior to alternative methods is not made. Rather, it is proposed that, as different methods will be more appropriate in different circumstances. No method of health-state valuation is perfect, and so inevitably researchers must make pragmatic decisions.

DCE-VAS does not require trading life-years. This can be useful if studying populations for which it is reasonable to assume there will be a reluctance to sacrifice length for quality of life. In addition, it imposes a low respondent burden and is possible to administer remotely, which may have practical advantages. On the other hand, if it is desirable to embed an explicit notion of opportunity cost into the tasks participants perform, then a method such as DCE-TTO or a hybrid model would be preferred.

## Footnotes

1 https://euroqol.org/eq-5d-instruments/eq-5d-5l-about/valuation-standard-value-sets/ accessed 22/11/18.
